# Cardiac Autonomic Dysfunction in Myasthenia Gravis and Relapsing-Remitting Multiple Sclerosis—A Pilot Study

**DOI:** 10.3390/jcm10102173

**Published:** 2021-05-18

**Authors:** Łukasz Rzepiński, Monika Zawadka-Kunikowska, Julia L. Newton, Paweł Zalewski

**Affiliations:** 1 Department of Neurology, 10th Military Research Hospital and Polyclinic, Powstańców Warszawy 5, 85-681 Bydgoszcz, Poland; 2 Department of Hygiene, Epidemiology, Ergonomy and Postgraduate Education, Ludwik Rydygier Collegium Medicum in Bydgoszcz Nicolaus Copernicus University in Torun, M. Sklodowskiej-Curie 9, 85-094 Bydgoszcz, Poland; m.zkunikowska@cm.umk.pl (M.Z.-K.); p.zalewski@cm.umk.pl (P.Z.); 3 Population Health Science Institute, The Medical School, Newcastle University, Framlington Place, Newcastle-upon-Tyne NE2 4HH, UK; julia.newton@newcastle.ac.uk

**Keywords:** myasthenia gravis, relapsing-remitting multiple sclerosis, heart rate variability, blood pressure variability, sympathovagal ratio, cardiac autonomic dysfunction

## Abstract

This study assessed cardiac autonomic response to head-up tilt test (HUTT) in 23 myasthenia gravis (MG) and 23 relapsing-remitting multiple sclerosis (RRMS) patients compared to 30 healthy controls (HC). Task Force^®^ Monitor was used to evaluate cardiac inotropy parameters, baroreflex sensitivity (BRS), heart rate (HRV), and blood pressure variability (BPV) during HUTT. MG patients were characterized by reduced BRS (*p* < 0.05), post-HUTT decrease in high-frequency component (*p* < 0.05) and increase in sympathovagal ratio of HRV (*p* < 0.05) when compared to controls indicating parasympathetic deficiency with a shift of sympathovagal balance toward sympathetic predominance. Compared to HC, MG patients also showed lower cardiac inotropy parameters, specifically, left ventricular work index (LVWI) during supine rest (*p* < 0.05) as well as LVWI and cardiac index values in response to orthostatic stress (*p* < 0.01 and *p* < 0.05, respectively). Compared to controls, RRMS patients were characterized by lower HRV delta power spectral density (*p* < 0.05) and delta low-frequency HRV (*p* < 0.05) in response to HUTT suggesting combined sympathetic and parasympathetic dysfunction. There were no differences in cardiac autonomic parameters between MG and MS patients (*p* > 0.05). Our study highlights the possibility of cardiac and autonomic dysfunction in patients with MG and RRMS which should be considered in the pharmacological and rehabilitation approach to managing these conditions.

## 1. Introduction

Multiple sclerosis (MS) and myasthenia gravis (MG) are the most common acquired autoimmune disorders targeting the central nervous system (CNS) and the neuro-muscular junction (NMJ), respectively [1,2]. Both diseases are caused by dysfunctions in self-tolerance in response to over activity of the immune system. MG is the best understood autoantibody mediated neurological disease whilst MS pathogenesis is not fully understood but involves abnormal responses of autoreactive T and B lymphocytes in the CNS. The immune component is the leading cause of clinical deterioration in MG throughout course of the disease, whereas in MS it is mainly seen during the relapsing-remitting phase (RRMS) [3,4,5]. Nevertheless, MG and MS share many similarities, such as a genetic predisposition, early and late disease onset with comparable gender distribution, a relapsing or progressive course with a wide spectrum of clinical symptoms, and favorable response to anti-CD20 monoclonal antibody as well as various other forms of immunosuppressive therapies [3,4,6]. The predominant MG manifestation is caused by autoantibodies that bind to the acetylcholine receptors (AChR) or to functionally related postsynaptic membrane molecules of the NMJ resulting in cholinergic transmission impairment. It has been suggested that the cholinergic deficit in MG may affect other regions as well, making the disease a “neurologic chameleon” able to mimic RRMS symptoms [7,8,9].

Autonomic dysfunction (AD) has been observed in various neurological disorders and leads to unfavorable long-term clinical outcomes. Because of the reduced quality of life and the increased risk of cardiovascular events seen with AD, early and appropriate diagnosis of dysautonomia remains particularly important [10]. Assessment of the autonomic nervous system (ANS) can be performed based on patient-reported symptoms and laboratory findings. In the laboratory, the most frequently examined part of dysautonomia is cardiovascular autonomic dysfunction (CAD) because its high availability for evaluation [11]. Short-term spectral analysis of beat-to-beat blood pressure (BPV) and heart rate variability (HRV) is a valuable, non-invasive tool for CAD assessment [12].

Dysautonomia is not a commonly reported clinical feature of MG and MS. Although previous studies have shown ANS involvement in the course of both RRMS and MG, the results were inconsistent and highly dependent on disease activity [13,14]. Inflammation is a major driver of RRMS and MG pathology closely related to the activity and severity of both diseases. Therefore, both RRMS and MG patients have increased numbers of pro-inflammatory T-helper 1 (Th1) and Th17 cells along with their associated pro-inflammatory cytokines such as interleukin (IL)-1, IL-6, IL-17, interferon-γ, and tumor necrosis factor alpha (TNF-α) [1,15]. Moreover, in patients with MG exacerbation as well as MS relapse, a more pronounced shift in inflammatory cytokine networks towards a pro-inflammatory response is observed compared to those with a stable disease course [16,17]. The literature suggests the presence of a feedback loop between the autonomic and immune systems. Importantly, the immune cells express both adrenergic as well as cholinergic receptors, and acetylcholine is not only the principal neurotransmitter at the NMJ but also at the autonomic ganglia [18,19]. The basic neuroimmunomodulating loop was identified between the vagus nerve (VN) and immune system, and was defined as a cholinergic anti-inflammatory pathway. It has been shown that pro-inflammatory cytokines from the periphery signal the brain via the afferent VN, leading to fever and activation of stress response. Conversely, anti-inflammatory mechanisms are mediated by efferent VN. Pharmacological/electrical stimulation of VN leads to the release of acetylcholine which suppresses the production of TNF-α and other pro-inflammatory cytokines in lipopolysaccharide-stimulated human macrophage cultures [19,20,21]. Therefore, the modulation of vagal activity may result in beneficial therapeutic effects for both MG and MS patients [20,21]. Conversely, the cholinergic deficit observed in MG may contribute to the intensification of the autoimmune response. We hypothesize that the mechanisms underlying MG and MS pathogenesis, along with the cholinergic transmission disturbances observed in MG, may translate into a specific pattern of AD. To date, no studies comparing autonomic imbalance in MG and MS have been conducted. Thus, we aimed to compare the pattern of CAD in MG and MS patients in relation to healthy controls (HC). Due to the difficulties in reliably linking the clinical activity of both diseases, we evaluated only those MG and RRMS patients who had had at least a three-month period of stable disease course.

## 2. Materials and Methods

### 2.1. Study Participants and Study Protocol

The study was approved by the Bioethical Committee of Collegium Medicum in Bydgoszcz, Nicolaus Copernicus University in Torun (KB 747/2017) and was performed in accordance with the principles of the Declaration of Helsinki. All subjects participated voluntarily and gave their written informed consent. The study was conducted over a three-year period from November 2017 to November 2020. Inclusion criteria for patients were diagnosis of MG or RRMS and no disease exacerbation within 90 days preceding the study. Age-matched subjects without disorders of the central and peripheral nervous system served as HC. Patients and HC with a history of hypertension, cardiac ischemic heart disease, hyperthyroidism, hypothyroidism, diabetes mellitus, psychiatric disorders, tobacco, and alcohol abuse were excluded from the study. Furthermore, participants using antiarrhythmic or antihypertensive drugs, anticholinergic therapy, anxiolytics, antidepressants, and other medications affecting cardiovascular system, including MS patients on fingolimod, were not enrolled. Demographic and clinical data were derived from medical records.

The study population was divided into three groups. Study group 1 included 23 patients consulted in the neurological outpatient clinic with a diagnosis of MS according to the 2010 McDonald criteria [22] all were classified as relapsing-remitting MS (RRMS). RRMS was characterized by disease relapses followed by a complete or partial recovery, without disability progression between the bouts. A relapse was defined as an episode of new or worsening of previous MS-related symptoms lasting over 24 h in the absence of fever and/or infection. Neurological deficits occurring within one month were considered as a single relapse [23]. Disability of patients was determined in accordance with the Kurtzke Expanded Disability Status Scale (EDSS), a 10-point disease severity score ranging from 0 (normal neurological exam) to 10 (death due to MS) in 0.5 increments [24]. Scores from 0 to 4.0 indicate at least 500 m of ambulation without assistance, while scores greater than or equal to 4.5 indicate a progressive reduction in gait capability as well as type of assistance required [25].

Study group 2 comprised 23 MG patients treated in neurological outpatient clinic. MG was diagnosed on the basis of clinical features (fluctuating weakness of ocular and/or extraocular muscles) and at least one of the following criteria: positive test for AChR or muscle-specific kinase (MuSK) autoantibodies, electrophysiologcal studies confirming postsynaptic neuromuscular junction dysfunction (repetitive stimulation and/or single fiber electromyography), and clinical response to cholinesterase inhibitors. With regard to the clinical disease type, ocular MG (symptoms restricted to the ocular muscles) or generalized MG (confirmed involvement of extraocular muscles) were distinguished [3]. Disease severity was assessed using the Myasthenia Gravis Foundation of America (MGFA) classification separating patients into five groups. MGFA classes involve pure ocular weakness (class I), mild-generalized weakness (class II), moderate-generalized weakness (class III), severe-generalized weakness (class IV), and intubation with or without mechanical ventilation (class V). Within the generalized MG types, patients are subcategorized as class A (predominant limb/axial muscles involvement) or class B (predominant oropharyngeal/respiratory muscles involvement) [26]. MG exacerbation was defined as the presence of new or worsening of previously reported muscle weakness lasting more than 24 h unrelated to fever and/or infection resulting in an increase in the MGFA classification by at least one class. The worsening of symptoms within 30 days were considered as a single exacerbation. The thymic abnormalities were assessed based on CT scan results and available histology findings. All MG patients were tested for the presence of serum anti-AChR antibodies by an enzyme-linked immunosorbent assay (ELISA). The values greater than or equal to 0.4 nmmol/L were considered abnormal. IgG4 antibodies against MuSK were measured by ELISA in subjects lacking anti-AChR antibodies.

Study group 3 consisted of 30 HC recruited from the community of Bydgoszcz in northern Poland.

The evaluation of patients in the MGFA classification and EDSS was performed on the day of the autonomic function tests. In the HC group, only autonomic functions were tested.

### 2.2. Cardiac and Autonomic Measures

All measurements were performed at the same time of the day, between 8–12 a.m., in a quiet, darkened room with standard temperature (22 ± 1 °C) and air humidity, by the same investigator. All study participants were asked to refrain from alcoholic and caffeinated beverages, smoking, and exercise for at least 12 h prior to the study, but were permitted to drink (water, juice) and to take their medicines [27,28].

The protocol included 15 min of baseline rest (supine), followed by 5 min of head up tilt test at 70 degrees. Cardiac and autonomic functioning of participants was recorded noninvasively using the validated Task Force^®^ Monitor (TFM, CNSystems, Graz, Austria). TFM integrates an electrocardiogram (ECG), oscillometric, continuous plethysmographic blood pressure registration, impedance cardiography (ICG), allowing analysis of the power spectral analysis of heart rate variability (HRV), and blood pressure variability (BPV) via adaptive autoregressive model (AAR) [29,30]. Heart rate (HR) was calculated from six-lead ECG, while the ICG was used to evaluate stroke index (SI = SV/body surface), cardiac index (CI = CO/body surface), thoracic fluid content (TFC), left ventricular ejection, time (LVET) and left ventricular work index (LVWI), representing cardiac inotropy. The total peripheral resistance index (TPRI) was calculated according to Ohm’s law: total peripheral resistance index = mean BP/cardiac index [31]. A beat-to-beat BP was also recorded using the finger cuff attached to the right second and third finger. The Task Force Monitor’s oscillometric blood pressure cuff was placed on the left upper arm [29]. TFM calculates total power spectral density (PSD) and three main frequency bands, defined as: very-low frequency (VLF), low-frequency (LF), and high-frequency (HF), but only two of these were taken account of as there were short-term autonomic regulations of beat-to-beat HR and BP signals. LF band (LF 0.05–015 Hz) and HF band (HF 0.15–0.4 Hz) were expressed in absolute values (ms2) and normalised units (nu): LFnu-RRI, HFnu-RRI for heart rate variability and LFnusBP, HFnu-sBP, LFnu-dBP, and HFnu-dBP for systolic and diastolic blood pressure variability [32]. Frequency-domain parameters—such as PSD, LF, and HF—are considered to be reliable markers of the cardiac autonomic regulation [33]. PSD is the power distribution across frequencies, representing total variability. LF band is considered to measure both sympathetic and parasympathetic modulation of the sinotrial node and vasomotor function, HF band solely parasympathetic modulation of cardiac activity and the ratio between LF and HF bands (LF/HF) reflect the sympathovagal balance [34,35]. We also measured baroreceptor sensitivity (BRS) using spontaneous sequence method as the slope of the linear regression between beat-to-beat sBP values (mmHg) [33].

### 2.3. Statistical Analysis

HRV and BPV data, were exported from the TFM software version 2.3.20.20 (TFM, CNSystems Medizintechnik, Graz, Austria) to MS-Excel (version 2019, Microsoft 365 Personal, Poland) for further preparation and then transferred to Statistica (version 13.3, StatSoft, Poland) for statistical analysis. AAR model may produce outliers when analysing RR intervals, thus all HR beat-to-beat data was filtered using Grubbs’ test for outlier elimination. This method of filtering is well-documented and has a strong mathematical background [36]. The results are presented as mean ± standard deviation (SD). Normal distribution of the study variables was verified with the Shapiro–Wilk test. Differences in the distribution of qualitative variables were determined with the χ2 test, while the differences in quantitative variables were determined with the use of a parametric *t*-test or a non-parametric Mann–Whitney test. Multiple comparisons were performed by one-way analysis of variance (ANOVA), followed by Tukey’s HSD test or by the Kruskal–Wallis rank sum test. A strength and significance of correlation between selected variables were calculated using the nonparametric Spearman’s test. The level of significance for all tests was set at *p* < 0.05.

## 3. Results

The demographic and clinical characteristics of the study population are presented in Table 1. There were no significant differences between the MG Group and MS Group with regard to the patients’ age, gender and disease duration (*p* > 0.05). Thymic pathology was detected in 13 (56.5%) MG patients and nine of them underwent thymectomy. The histopathological evaluation revealed thymic hyperplasia in eight cases and thymoma in one case. In MG group, 6 (26.1%) patients were double-seronegative (negative test for anti-AChR and anti-MuSK antibodies). No patient with generalized MG had predominant oropharyngeal or respiratory muscle involvement. At the time of the study, all MG patients were on pyridostigmine at an average dose of 180 mg/day, 10 of them used corticosteroids and 9 required immunosuppressive therapy (6 azathioprine and 3 mycophenolate mofetil). A total of 10 MS patients (43.5%) received immunomodulatory drugs (IMDs): seven interferon-beta, two glatiramer acetate and one natalizumab.

### 3.1. Clinical Symptoms of Autonomic Imbalance

The incidence of clinical signs of AD in patients with MG and MS is presented in Table 2. No patient from the HCs Group reported symptoms of dysautonomia. MG patients experienced a significantly higher frequency of dry mouth/eyes, sudden paleness, thermoregulatory disorders, anxiety (*p* < 0.05) and diarrhoea (*p* < 0.01), as compared to the MS Group. None of the examined participants showed abnormal HR in response to standing that fulfilled the positional tachycardia syndrome (POTS) criteria. Only one MS patient (4.3%) experienced orthostatic hypotension (OH) symptoms.

### 3.2. Hemodynamic Parameters Assessment

**Figure 1:**
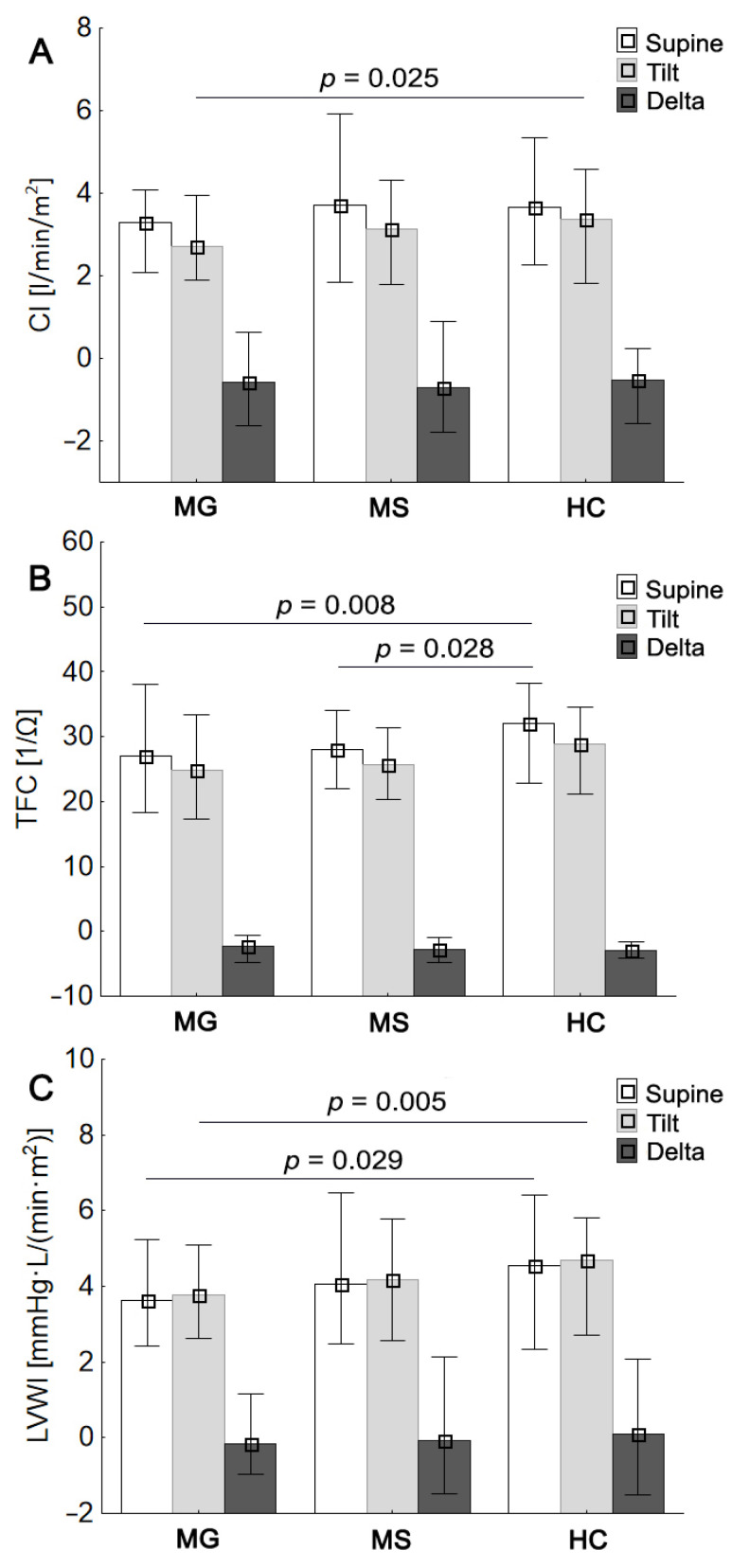
MG, MS, and HC groups mean values (±SD) of CI, cardiac index (**A**); TFC, thoracic fluid content (**B**); LVWI, left ventricular work index (**C**); respectively for compared to healthy controls.

All enrolled participants underwent the study protocol. During the autonomic tests, all participants had sinus heart rhythm. The values of HR, BP, SI, and TPRI were comparable between the evaluated groups, both at rest and during the head-up tilt test (HUTT) (*p* > 0.05; Table 3). Compared to controls, MG patients had significantly lower parameters associated with myocardial contractility, including LVWI values during supine rest (*p* < 0.05) as well as LVWI and CI values in response to orthostatic stress (*p* < 0.01 and *p* < 0.05, respectively) (Figure 1A,C). The decrease in myocardial contractility parameters in MG group did not translate into TFC values, which turned out to be significantly lower in MG subjects with respect to HCs ones (Figure 1B). Compared to HC, RRMS patients showed lower TFC values (*p* < 0.05) without a decrease in myocardial contractility parameters (Figure 1A–C). No significant differences were observed between the MG group and RRMS group for analyzed hemodynamic parameters, both at rest and after HUTT (*p* > 0.05; Table 3).

### 3.3. Baroreflex Sensitivity, Heart Rate, and Blood Pressure Variability Analysis

**Figure 2:**
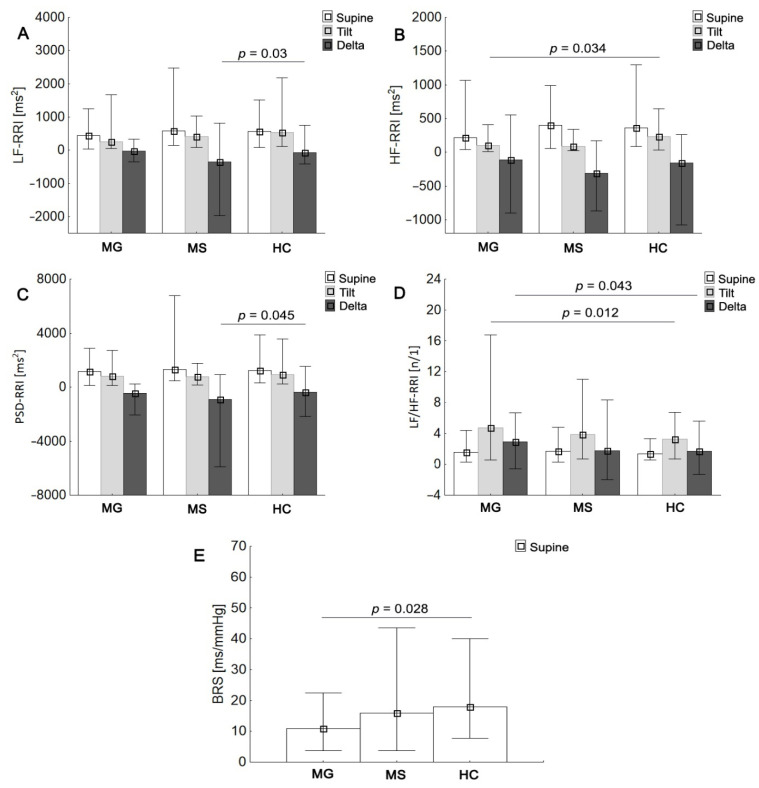
MS and MG groups mean values (±SD) of LFnu-RRI, low frequency R-R interval in normalised units (**A**); HFnu-RRI, high frequency R-R interval in normalised (**B**); PSD-RRI, power spectral density of heart rate variability (**C**); LF/HF-RRI, ratio between low and high band for heart rate variability (**D**); BRS, baroreflex sensitivity (**E**); respectively for compared to healthy controls.

At rest, no significant intergroup differences were found in HRV, BPV, and sympathetic–parasympathetic balance (LF/HF ratio) (*p* > 0.05; Table 4). With respect to HC, MG patients showed lower HF-RRI component (*p* < 0.05) and higher LF/HF-RRI ratio (both absolute and delta values, *p* < 0.05) in response to orthostatic stress as well as reduced baroreflex sensitivity (*p* < 0.05), all suggesting parasympathetic deficiency with a shift of sympathovagal balance toward sympathetic predominance (Figure 2). Compared to the controls, RRMS patients were characterized by significant decrease in HRV parameters (delta PSD-RRI, *p* < 0.05 and delta LF-RRI, *p* < 0.05) in response to HUTT (Figure 2). There were no intergroup differences in BPV components during orthostatic stress (*p* > 0.05). Baroreflex sensitivity and post tilting HRV parameters recorded in MS group were not significantly different from the values obtained in MG and HC groups (*p* > 0.05).

### 3.4. Association of Cardiovascular and Autonomic Measures with MS Clinical Outcomes

Correlation analyses revealed a significant association of RRMS duration with sBP (*r* = −0.42, *p* = 0.046) and mBP (*r* = −0.44, *p* = 0.034). The EDSS score was inversely correlated with cardiac parasympathetic components of BPV—i.e., HFnu-dBP (*r* = −0.42, *p* = 0.044), HFnu-sBP (*r* = −0.58, *p* = 0.004), HF-sBP (*r* = −0.43, *p* = 0.04), and positively correlated with LF/HF-sBP ratio (*r* = 0.46, *p* = 0.029). The age of the first RRMS symptoms was positively associated with LVET (*r* = 0.61, *p* = 0.002) and delta HF-sBP (*r* = 0.43, *p* = 0.40), whereas negative correlations of this parameter with sympathovagal balance measures in the form of delta LF/HF-RRI (*r* = −0.42, *p* = 0.043), delta LF/HF-sBP (*r* = −0.47, *p* = 0.023), and delta LF/HF (*r* = −0.45, *p* = 0.031) were found.

### 3.5. Association of Cardiovascular and Autonomic Measures with MG Clinical Outcomes

Age of MG onset was positively correlated with dBP (*r* = 0.46, *p* = 0.028), MBP (*r* = 0.47, *p* = 0.023), and sympathetic overactivation parameters such as delta LF/HF-sBP (*r* = 0.38, *p* = 0.028), LFnu-sBP (*r* = 0.44, *p* = 0.035), LF-sBP (*r* = 0.47, *p* = 0.024), and TPRI (*r* = 0.41, *p* = 0.049). Furthermore, the disease severity as determined by the MGFA classification was positively associated with sympathovagal balance parameters in the form of delta LF/HF-RRI (*r* = 0.63, *p* = 0.001) and delta LF/HF (*r* = 0.62, *p* = 0.002) as well as negatively correlated with baroreceptors sensitivity (*r* = −0.51, *p* = 0.012) and TFC (*r* = −0.44, *p* = 0.038).

All the performed correlations of cardiovascular and autonomic measurements with MG and MS clinical data are presented in Supplementary Materials (Tables S1 and S2).

## 4. Discussion

In this study, we aimed to compare CAD in MG and RRMS patients not influenced by fluctuations in disease course and in relation to HC. Overall, we assessed autonomic function in 76 study participants with a similar age distribution. The MG and RRMS patients had comparable age of first clinical manifestation and duration of the disease. Our findings showed different patterns of cardiovascular autonomic dysfunction in the MG and MS groups when compared to HC ones, whereas no significant differences of cardiac modulation parameters between MG and MS were found. The main finding of this study was parasympathetic deficiency (HF-RRI) with a shift of sympathovagal balance toward sympathetic predominance in the MG Group when compared to the controls. Furthermore, MG patients showed significantly reduced parameters of cardiac inotropy when compared to HC and a significantly higher incidence of AD symptoms with respect to MS patients. Conversely, RRMS patients showed no decline in myocardial contractility parameters and were more predisposed to combined sympathetic and parasympathetic dysfunction during HUTT. However, for both MG severity and the disability in MS course, a positive correlation with hyper-reactivity of the sympathetic nervous system was found. These findings appear to be relevant for several reasons. Firstly, the initial pattern of autonomic system damage in MG and RRMS seems to evolve in different directions compared to controls. Secondly, MG patients are at increased risk of cardiac conduction disturbances compared to healthy peers due to sympathetic overactivity and impaired baroreflex mechanism [37]. Thirdly, the symptoms of dysautonomia need to be considered in the routine evaluation of MG and MS patients.

Contrary to the evaluation of patients with movement disorders, the routine diagnostic panel of MG and MS does not include the assessment of the autonomic system. The signs of dysautonomia may be a consequence of the disease course, comorbidities or the type of treatment applied. In our group, there were no significant differences in the frequency of experiencing cardiac arrhythmia between MG and MS patients, which was consistent with the subsequent CAD testing. Nevertheless, MG patients significantly more frequently reported non-cardiac symptoms of autonomic imbalance than those with MS. Some of these signs could be related to parasympathetic deficiency (dry mouth), while others were highly likely to be associated with drugs (e.g., diarrhea—pyridostigmine or anxiety—corticosteroids). A study by Nikolić et al. revealed that MG patients rarely complain of autonomic symptoms, but when asked and examined for them, some clinical signs of dysautonomia can be found in the majority of these patients [38]. Therefore, screening for autonomic dysfunction should be recommended in all patients diagnosed with MG or MS at various stages of the disease, including the pre-treatment period. Due to the lack of data on this issue in the literature, it was not possible to compare our results with other studies.

Among the symptoms of dysautonomia, those associated with the cardiovascular system are the most life-threatening. In the current study, we showed intergroup differences in the parameters of short-term cardiac modulation between young participants without diagnosed cardiovascular disease. Compared to the controls, the initial pattern, evolution and degree of changes in cardiac modulation turned out to be different in MG group than in MS group. MG patients were characterised by significantly lower myocardial inotropic function variables, more pronounced after HUTT than at rest. Furthermore, in response to orthostatic stress, they experienced a decrease in the parasympathetic tone and shift of the sympathovagal balance towards sympathetic tone in terms of HRV. Cardiac involvement in MG has been reported and ranged from asymptomatic ECG findings through conduction abnormalities and heart failure to sudden death [39,40]. Overall, 12% of MG patients may experience various clinical signs of cardiac disease. Moreover, nearly half of MG patients have antistriatal antibodies (e.g., against titin, ryanodine receptor, or voltage-gated potassium channel) that can bind to the heart muscle causing myocarditis [41,42]. Generally, patients with late-onset (≥50 years of age) or thymoma associated MG were considered to be the risk group for cardiovascular events [42]. However, later reports have described cardiac dysfunction in patients with early onset of MG and without thymic pathology which improved after pirydostygmine and/or steroids [43,44]. Our findings support the presence of heart involvement in early-onset MG patients who were treated with pyridostigmine and showed a stable disease course within the last three months preceding the evaluation. Benjamin et al. have shown autonomic dysfunction in myasthenic crises involving multiple organ systems that may be accounted for by the parasympathetic deficiency [45]. Furthermore, the results of the study by Kaltsatou et al. have supported the hypothesis of central cholinergic effects of MG manifested by cognitive dysfunction [7]. In the context of these reports, as well as our findings, it can be presumed that the cholinergic deficit in MG drives beyond the striated muscles and may contribute to the shift of cardiac sympathovagal balance towards sympathetic hyperresponsiveness.

Several previous studies have revealed sympathovagal imbalance in favor of sympathetic tone and decreased baroreflex in MG patients, which is in line with our results [13,38,46]. This sympathetic dominance could have resulted in significantly lower TFC values in MG patients than in the controls despite the decrease in the myocardial contractility parameters. The shift towards sympathetic hyperresponsiveness in MG is secondary to parasympathetic insufficiency and, apart from a disturbed reflex from baroreceptors, may be a result of impaired synaptic transmission in autonomic ganglia through the cross reactivity of muscular anti-AChR Ab with ganglionic AChR [44,47]. It has been suggested that other NMJ postsynaptic molecules such as MUSK, low-density lipoprotein receptor-related protein 4 (LRP4) and agrin may interfere with the formation and plasticity of CNS synapses [48,49,50]. Thus, antibodies against these targets may induce an immune response with autonomic ganglia antigens cross reactivity, contributing to AD in MUSK-positive or double seronegative MG patients [38]. In the presented study, spectral domain HRV parameters showed both sympathetic predomination and lower vagal tone in response to orthostatic stress which confirm the above assumptions. These findings, combined with impaired baroreflex mechanism, are known to predispose to cardiac conduction abnormalities and sudden death [32]. Importantly, we found a positive association of MG severity with sympathetic nervous system hyperresponsiveness and lower BRS. Therefore, the risk of severe cardiac abnormalities is highest in patients during myasthenic crisis, which is consistent with the results reported by other authors [13,44]. Nevertheless, the occurrence of CAD in the stable disease course should be considered, more so as exercise intolerance commonly reported by MG patients may lead to the underdiagnosis of comorbid cardiac disorders.

In the present study, RRMS patients showed an evolution from a deficit of combined sympathetic and parasympathetic response to orthostatic stress when compared to HC towards sympathetic dominance along with the disability progression. It has been suggested that dysautonomia may enhance inflammatory and neurodegenerative mechanisms in MS contributing to disability progression [51]. Autonomic imbalance in RRMS may be attributed to demyelinating lesions within the CNS involved in the modulation and control of the ANS through catecholamine release and induction of β-adrenergic receptors expression in peripheral blood mononuclear cells [51]. Flachenecker et al. found that sympathetic dysfunction was associated with the clinical activity of RRMS, while parasympathetic dysfunction was closely related to disability progression [14]. A recent review article confirmed interactions between the autonomic and immune systems in the course of MS, suggesting sympathetic nervous system dysfunction in the early RRMS phase and parasympathetic nervous system dysfunction in advanced progressive MS types [11]. In a study by Studer et al., the evaluation of HRV revealed defective sympathetic tone in RRMS patients, even with subclinical disease activity, whereas stable RRMS patients did not differ from healthy controls. Furthermore, the overactivity of the sympathetic system was more evident among severely disabled patients and was correlated with irreversible disability [52]. In our study, we did not analyze the subclinical (radiological) activity of RRMS, which makes it difficult to reliably relate our findings to the results obtained by Studer et al. However, the correlation of disability progression in RRMS patients with the parasympathetic insufficiency and the combined sympathetic-parasympathetic dysfunction after HUTT found in this group is consistent with the above-mentioned reports.

The immune system plays a major pathogenic role in both MG and MS, leading to patients’ disability [1]. The VN stimulation suppress inflammation via cholinergic anti-inflammatory pathway. The cholinergic system has been suggested as a mediator between the nervus and immune systems and as a modulator of immune responses [20,21]. Thus, VN modulation may alleviate autoimmune disorders including MG and MS through acetylcholine release. Marrosu et al. have shown that VN stimulation reduces cerebellar tremor and dysphagia in MS patients [53]. Moreover, the available data based on case reports suggest a beneficial effect of VN stimulation through slow breathing sessions as well as chiropractic therapy in reducing MG symptoms [54,55,56]. Furthermore, Antonino et al. have demonstrated that VN stimulation acutely improves spontaneous cardiac baroreflex sensitivity in healthy young men [57]. In the context of these data, both the decreased vagal tone and disturbed reflex from baroreceptors seem to be closely related to the function of the VN. Therefore, vagally-mediated anti-inflammatory pathway should be considered as potential therapeutic approach for MG and MS. Further tests are needed to precisely assess the effectiveness and indications for VN stimulation in patients with MG and MS.

We propose that parasympathetic deficiency may in fact have preceded MG and its cardiovascular abnormalities, both related to insufficient vagal modulation of inflammation. This hypothesis is more plausible given that our patients had stable disease course but still showed vagal abnormality correlated with the severity of MG and MS. However, due to the cross-sectional study design, these considerations should be interpreted with caution.

A few limitations of our study should be mentioned. First, the investigated cohort is relatively small, which made it impossible to carry out some statistical analyzes, e.g., the multiple regression model. Moreover, despite the use of Tukey’s test for multiple comparisons, the vast number of statistical tests and the little number of significant findings cannot at all rule out chance results and a type-1 error. Future studies should use more focused and a priori statistical tests taking into account our preliminary results. Thus, our findings need further evaluation on a larger sample size. Second, we did not assess the antibody titer against striated muscles, agrin and LRP 4 which could more fully explain the involvement of the autonomic system in MG patients. In turn, MS patients did not have assessment of radiological disease activity. Therefore, we were unable to correlate the number and activity of demyelinating plaques with the severity of autonomic imbalance. Although MS patients were clinically stable in the three months preceding the study, we cannot rule out radiological disease activity at that time. The implementation of various therapeutic agents, especially in MG patients—along with the above-mentioned limitations—did not allow us to assess the influence of the drugs used on autonomic dysfunction.

## 5. Conclusions

Our study shows a different pattern of autonomic nervous system involvement in MG and MS patients with a comparable mean disease duration and a stable three-month clinical course. Patients with MG were characterized by more frequent occurrence of autonomic symptoms compared to those with MS and reduced cardiac inotropy parameters as well as parasympathetic insufficiency in response to orthostatic stress when compared to the controls. In contrast, RRMS patients were characterized by combined sympathetic and parasympathetic dysfunction in response to HUTT compared to HC. There were no differences in cardiac autonomic parameters between MG and MS patients. However, both diseases showed a positive correlation between disease severity and sympathetic hyperresponsiveness. Our study highlights the possibility of autonomic dysfunction in patients with MG and RRMS despite the stable disease course, which should be widely considered in the pharmacological and rehabilitation approach. To the best of our knowledge, this is the first study comparing autonomic impairment between these conditions.

## Figures and Tables

**Table 1:** Demographic and clinical data of the studied patients

	MG	RRMS	HC	*p*
Number of subjects (*n*)	23	23	30	
Sex (Male/Female)	4/19	1/22	6/24	0.246
Age, years	40.6 ± 11.0	39.9 ± 10.1	36.2 ± 7.7	0.199
Disease duration (years) (range)	8.4 ± 7.8 (0–24)	7.7 ± 5.7 (0.5–23)		0.732
Age of first symptoms (years) (range)	32.2 ± 12.1 (12.0–59.0)	32.2 ± 9.4 (20.0–58.0)		0.989
Type of MG				
Ocular	6 (26.1%)			
Generalized	18 (78.3%)			
Seropositivity to AChR antibodies, *n* (%)	17 (73.9%)			
Seropositivity to MuSK antibodies	0 (0%)			
Double-seronegative MG	6 (26.1%)			
Thymic pathology (%)	13 (56.5%)			
Thymectomy, *n* (%)	9 (39.1%)			
Thymoma	1 (4.3%)			
Disease stage (MGFA), *n* (%)				
Class I	6 (26.1%)			
Class IIa	11 (47.8%)			
Class IIIa	6 (26.1%)			
Type of treatment				
Cholinergic therapy	23 (100%)			
Corticosteroids	10 (43.5%)			
Immunosuppressive therapy	9 (39.1%)			
IMDs		10 (43.5%)		
Mean EDSS score (range)		2.1 (0.5–6.0)		

Abbreviations: MG—myasthenia gravis; RRMS—relapsing-remitting multiple sclerosis; HCs- healthy controls; AchR—acetylcholine receptor; MuSK—muscle-specific kinase; MGFA—Myasthenia Gravis Foundation of America classification; EDSS—Expanded Disability Status Scale; IMDs—immunomodulatory drugs.

**Table 2:** Clinical symptoms of autonomic dysfunction.

Non-Motor Symptoms	MG		RRMS		*p*-Value
	Present	Absent	Present	Absent	
	*n* (%)	*n* (%)	*n* (%)	*n* (%)	
Orthostatic disorders	13 (56.5%)	10 (43.5%)	17 (73.9%)	6 (26.1%)	0.215
Dizziness	14 (60.9%)	9 (39.1%)	17 (73.9%)	6 (26.1%)	0.345
Sudden paleness	11(47.8%)	12 (52.2%)	3 (13.0)	20 (87.0%)	0.013 *
Arrhythmia episodes	10 (43.5%)	13 (56.5%)	5 (21.7%)	18 (78.3%)	0.115
Vasomotor disorder	4 (17.4%)	19 (82.6%)	2 (8.7%)	21 (91.3%)	0.381
Dry mouth/eyes	16 (69.6%)	7 (30.4%)	9 (39.1%)	14 (60.9%)	0.025 *
Thermoregulatory disorders	15 (65.2%)	8 (34.8%)	6 (26.1%)	17 (73.9%)	0.010 *
Stomach ache	7 (30.4%)	16 (69.6%)	3 (13.6%)	19 (86.4%)	0.175
Constipation	8 (34.8%)	15 (65.2%)	5 (21.7%)	18 (78.3%)	0.325
Diarrhoea	9 (39.1%)	14 (60.9%)	0 (0%)	23 (100%)	0.001 **
Post-meal symptoms	11 (47.8%)	12 (52.2%)	9 (39.1%)	14 (60.9%)	0.551
Urinary bladder dysfunctions	10 (43.5%)	13 (56.5%)	5 (21.7%)	18 (78.3%)	0.115
Sexual dysfunction	6 (26.1%)	17 (73.9%)	4 (17.4%)	19 (82.6%)	0.474
Pupillary disorders	13 (56.5%)	10 (43.5%)	11 (47.8%)	12 (52.2%)	0.554
Anxiety	8 (34.8%)	15 (65.2%)	2 (8.7%)	21 (91.3%)	0.031 *

Abbreviations: MG—myasthenia gravis; RRMS—relapsing-remitting multiple sclerosis; statistically significant differences for compared to healthy controls are indicated with * *p* < 0.05, ** *p* < 0.01.

**Table 3:** Mean ± SD of resting and during tilt test haemodynamic parameters for patients with MG, MS, and healthy controls (HC)

Parameter	Supine			Tlt			Delta (Change Baseline-Tilt)		
	MG	MS	HC	MG	MS	HC	MG	MS	HC
HR (*n*/1)	64.6 ± 1.7	64.4 ± 1.6	66.8 ± 1.5	76.8 ± 2.3	78.4 ± 2.2	81.4 ± 1.9	12.2 ± 1.5	13.9 ± 1.5	14.6 ± 1.3
sBP (mmHg)	112.8 ± 2.6	109.8 ± 2.4	111.9 ± 2.2	123.6 ± 2.8	124.1 ± 2.6	125.0 ± 2.4	10.7 ± 2.5	14.1 ± 2.4	13.1 ± 2.2
dBP (mmHg)	71.6 ± 1.8	69.4 ± 1.7	73.4 ± 1.6	86.9 ± 2.3	89.4 ± 2.2	88.7 ± 2.0	15.3 ± 1.9	19.4 ± 1.8	15.3 ± 1.6
mBP (mmHg)	88.7 ± 2.0	86.5 ± 1.9	90.1 ± 1.8	101.8 ± 2.3	104.1 ± 2.2	104.1 ± 2.0			
SI (mL/m^2^)	51.4 ± 2.9	58.3 ± 2.7	56.3 ± 2.5	37.9 ± 1.5	39.6 ± 1.4	41.0 ± 1.3	−13.5 ± 2.2	−18.1 ± 2.1	−15.3 ± 1.9
CI (l/min/m^2^)	3.3 ± 0.2	3.7 ± 0.2	3.7 ± 0.2	2.9 ± 0.1 *	3.1 ± 0.1	3.3 ± 0.1	−0.4 ± 0.2	−0.6 ± 0.2	−0.4 ± 0.1
TPRI (dyn·s·m^2^/cm^5^)	2270.8 ± 147.5	1981.6 ± 141.0	1977.5 ± 127.8	2885.1	2790.3	2600.5	614.3 ± 136.7	752.4 ± 130.6	623.0 ± 118.4
LVWI (mmHg·L/min·m^2^)	3.8 ± 0.2 *	4.2 ± 0.2	4.5 ± 0.2	3.8 ± 0.2 **	4.3 ± 0.2	4.6 ± 0.2	0.1 ± 0.2	0.1 ± 0.2	0.1 ± 0.2
LVET (ms)	315.9 ± 2.8	323.0 ± 2.8	318.4 ± 2.5	284.6 ± 3.8	286.2 ± 3.8	279.5 ± 3.3	−31.2 ± 3.3	−36.7 ± 3.3	−38.9 ± 2.9
TFC (1/Ω)	27.9 ± 1.2 *	28.3 ± 1.4 *	32.7 ± 1.2	25.7 ± 1.3	25.8 ± 1.3	29.9 ± 1.1	−2.2 ± 0.2	−2.6 ± 0.2	−2.8 ± 0.2

Abbreviations: MS—multiple sclerosis; MG—myasthenia gravis; HC—healthy controls; HR—heart rate; sBP—systolic blood pressure; dBP—diastolic blood pressure; mBP mean blood pressure; SI—stroke index; CI—cardiac index; TPRI—total peripheral index; LVWI—left ventricular work index; LVET—left ventricular work index; TFC—thoracic fluid content, statistically significant differences for compared to healthy controls are indicated with * *p* < 0.05, ** *p* < 0.01.

**Table 4:** Mean ± SD of resting and during tilt test cardiac autonomic measures for patients with MG, RRMS, and HC

Parameter	Supine			Tlt			Delta (Change Baseline-Tilt)		
	MG	RRMS	HC	MG	RRMS	HC	MG	RRMS	HC
LFnu-RRI (%)	60.3 ± 3.3	59.5 ± 3.1	57.6 ± 2.8	75.5 ± 3.1	75.8 ± 3.1	72.2 ± 2.7	17.9 ± 3.2	15.6 ± 3.0	14.9 ± 2.7
HFnu-RRI (%)	39.7 ± 3.3	40.5 ± 3.1	42.4 ± 2.8	24.5 ± 5.8	24.2 ± 5.8	34.3 ± 5.1	−17.9 ± 6.2	−15.6 ± 5.9	−8.5 ± 5.2
LF-RRI (ms^2^)	593.0 ± 126.7	873.8 ± 121.1	726.4 ± 109.8	493.3 ± 186.0	486.5 ± 186.0	748.4 ± 162.8	−99.7 ± 115.6	−436.1 ± 110.4 *	28.3 ± 96.7
HF-RRI (ms^2^)	497.1 ± 160.2	751.0 ± 153.1	604.6 ± 138.8	166.8.1 ± 237.1 *	132.5 ± 237.1	266.1 ± 207.6	−330.3 ± 144.5	−621.5 ± 138.1	−338.3 ± 120.9
PSD-RRI (ms^2^)	2607.1 ± 675.2	2235.8 ± 645.2	1730.7 ± 584.8	1711.5 ± 460.5	805.2 ± 460.5	1285.1 ± 403.2	−896.4 ± 600.8	−1480.4 ± 574 *	−434.1 ± 503
LF/HF-RRI (*n*/1)	2.3 ± 0.4	2.0 ± 0.3	1.8 ± 0.3	6.2 ± 08 *	4.8 ± 0.8	3.9 ± 0.7	4.4 ± 0.7 *	2.5 ± 0.7	2.1 ± 0.6
LF/HF (*n*/1)	1.6 ± 0.3	1.4 ± 0.2	1.4 ± 0.2	4.1 ± 0.6	3.2 ± 0.6	2.8 ± 0.5	2.8 ± 0.5	1.6 ± 0.4	1.4 ± 0.4
LFnu-dBP (%)	44.5 ± 2.3	43.6 ± 2.2	47.0 ± 2.0	51.6 ± 2.7	51.8 ± 2.7	52.5 ± 2.4	7.6 ± 2.0	7.3 ± 2.0	6.0 ± 1.8
HFnu-dBP (%)	10.5 ± 1.0	10.7 ± 1.0	11.1 ± 0.9	12.8 ± 1.3	10.5 ± 1.3	11.8 ± 1.1	0.0 ± 0.6	−0.1 ± 0.6	0.8 ± 0.5
LF-dBP (mmHg^2^)	4.2 ± 0.6	3.9 ± 0.6	4.2 ± 0.5	3.8 ± 0.5	4.2 ± 0.5	3.4 ± 0.5	−0.7 ± 0.3	−0.3 ± 0.3	−0.6 ± 0.3
HF-dBP (mmHg^2^)	0.9 ± 0.2	1.0 ± 0.2	0.9 ± 0.1	1.1 ± 0.2	0.8 ± 0.2	0.8 ± 0.2	−0.4 ± 0.1	−0.3 ± 0.1	−0.1 ± 0.1
PSD-dBP (mmHg^2^)	9.3 ± 1.2	9.2 ± 1.1	8.8 ± 1.0	7.5 ± 1.0	8.1 ± 1.0	6.7 ± 0.8	−2.5 ± 0.5	−1.9 ± 0.5	−1.8 ± 0.5
LF/HF-dBP (*n*/1)	5.3 ± 0.5	4.8 ± 0.5	5.1 ± 0.5	5.4 ± 0.7	5.8 ± 0.7	5.5 ± 0.6	0.5 ± 0.5	0.8 ± 0.5	0.4 ± 0.4
LFnu-sBP (%)	41.3 ± 2.1	40.0 ± 2.0	41.8 ± 1.8	52.3 ± 2.8	51.8 ± 2.8	49.1 ± 2.4	11.0 ± 2.3	10.7 ± 2.3	8.5 ± 2.0
HFnu-sBP (%)	14.5 ± 1.7	11.9 ± 1.7	13.0 ± 1.5	18.1 ± 1.9	13.8 ± 1.9	15.0 ± 1.7	1.6 ± 1.0	2.1 ± 1.0	2.6 ± 0.8
LF-sBP (mmHg^2^)	6.7 ± 0.9	5.1 ± 0.9	5.5 ± 0.8	6.6 ± 1.1	6.4 ± 1.1	4.5 ± 0.9	−0.4 ± 0.5	−0.2 ± 0.5	−0.8 ± 0.4
HF-sBP (mmHg^2^)	2.1 ± 0.3	1.4 ± 0.3	1.7 ± 0.3	2.2 ± 0.3	1.3 ± 0.3	1.4 ± 0.3	−0.5 ± 0.2	−0.2 ± 0.2	−0.2 ± 0.2
PSD-sBP (mmHg^2^)	16.5 ± 2.1	13.4 ± 2.0	12.8 ± 1.9	12.3 ± 1.7	11.5 ± 1.7	9.2 ± 1.5	−4.7 ± 0.9	−3.6 ± 0.9	−3.4 ± 0.9
LF/HF-sBP (*n*/1)	4.0 ± 0.5	4.1 ± 0.4	4.0 ± 0.4	3.9 ± 0.5	5.0 ± 0.5	4.2 ± 0.5	2.8 ± 0.5	1.7 ± 0.5	1.5 ± 0.4
BRS (ms/mmHg)	12.7 ± 2.2 *	19.9 ± 2.1	19.9 ± 1.9						

Abbreviations: RRMS—relapsing-remitting multiple sclerosis; MG—myasthenia gravis; HC—healthy controls; LFnu-RRI—low-frequency R-R interval in normalized units; HFnu-RRI—high frequency R-R interval in normalized units; LF-RRI—low-frequency R-R interval; HF-RRI—high-frequency R-R interval; PSD-RRI—power spectral density R-R interval; LF/HF—ratio between low and high band for heart rate and blood pressure variability; LF/HF-RRI—ratio between low and high band for heart rate variability; LFnu-dBP—low frequency of diastolic blood pressure variability in normalized units; HFnu-dBP—high frequency of diastolic blood pressure variability in normalized units; LF-dBP—low frequency of diastolic blood pressure variability; HF-dBP—high frequency of diastolic blood pressure variability; PSD-dBP—power spectral density of diastolic blood pressure variability; LF/HF-dBP—ratio between low and high band for diastolic—blood pressure variability; LFnu-sBP—low frequency of systolic blood pressure variability in normalized units; HFnu-dBP—high frequency of systolic blood pressure variability in normalized units; LF-sBP—low frequency of systolic blood pressure variability; HF-sBP—high frequency of systolic blood pressure variability; PSD-sBP—power spectral density of systolic blood pressure variability; LF/HF-sBP—ratio between low and high band for systolic blood pressure variability; BRS—baroreflex sensitivity; nu—normalized values; statistically significant differences for compared to healthy controls are indicated with * *p* < 0.05.

## Data Availability

Not applicable.

## Supplementary Materials

The following are available online at https://www.mdpi.com/article/10.3390/jcm10102173/s1, Table S1: Association of cardiovascular and autonomic measures with RRMS and MG clinical; Table S2: Association of cardiovascular and autonomic measures with RRMS and MG clinical outcomes.
