# Supplementary material for: Cardiac Autonomic Dysfunction in Myasthenia Gravis and Relapsing-Remitting Multiple Sclerosis—A Pilot Study
Source: J Clin Med. 2021 May 18;10(10):2173. doi: 10.3390/jcm10102173 (PMC8157285; doi:10.3390/jcm10102173)
Supplement: Supplementary file 1 [file jcm-10-02173-s001.zip › jcm-1173893-supplementary.pdf]

Supplementary file

Table S1. Association of cardiovascular and autonomic measures with RRMS and MG clinical outcomes.

	**RRMS**			**MG**		
grupa MS	Disease duration	EDSS	Age of first symptom	Disease duration	MGFA	Age of first symptom
HR	−0.13	0.15	−0.32	0.37	0.32	−0.02
sBP	**−0.42** *	−0.02	0.22	−0.12	−0.24	0.26
dBP	−0.38	0.21	0.30	−0.10	0.10	**0.46** *
mBP	**−0.44** *	0.11	0.25	−0.13	−0.03	**0.47** *
SI	0.24	−0.26	0.04	0.02	−0.22	−0.36
CI	0.27	−0.06	−0.13	0.23	−0.05	−0.38
TPRI	−0.35	0.13	0.25	−0.10	0.07	**0.41** *
LVWI	0.07	0.02	0.05	0.03	−0.07	−0.10
LVET	0.07	−0.15	**0.61** **	−0.09	−0.29	0.30
TFC	0.30	−0.29	−0.16	0.17	**−0.44** *	−0.18
LFnu-RRI	0.30	0.02	−0.10	0.07	0.31	0.25
HFnu-RRI	−0.30	−0.02	0.10	−0.07	−0.31	−0.25
LF-RRI	0.18	0.00	−0.22	−0.22	−0.42	−0.15
HF-RRI	−0.08	−0.08	−0.05	−0.13	−0.30	−0.40
PSD-RRI	0.08	−0.01	−0.15	−0.05	−0.33	−0.35
LF/HF-RRI	0.31	0.02	−0.15	0.07	0.25	0.15
LF/HF	0.14	−0.08	−0.15	0.07	0.28	0.20
LFnu-dBP	0.00	−0.15	−0.11	0.03	0.18	0.16
HFnu-dBP	−0.33	**−0.42** *	**−0.43** *	0.21	−0.15	−0.28
LF-dBP	−0.16	−0.12	−0.06	−0.01	0.02	0.14
HF-dBP	−0.25	−0.30	−0.20	0.13	−0.05	−0.14
PSD-dBP	0.02	−0.07	−0.13	−0.08	−0.03	0.04
LF/HF-dBP	0.21	0.24	0.27	−0.10	0.25	0.25
LFnu-sBP	0.07	−0.18	0.05	−0.23	−0.23	**0.44** *
HFnu-sBP	−0.13	**−0.58** **	−0.25	−0.27	−0.15	−0.23
LF-sBP	−0.13	−0.08	0.19	0.04	−0.13	**0.47**
HF-sBP	−0.21	**−0.43**	−0.14	−0.08	−0.13	0.00
PSD-sBP	−0.08	0.00	0.08	0.13	−0.09	0.30
LF/HF-sBP	0.12	**0.46** *	0.29	0.08	0.10	0.38
BRS	0.06	0.13	−0.31	0.01	−0.09	**−0.51** *

HR, heart rate; sBP, systolic blood pressure; dBP, diastolic blood pressure; mBP mean blood pressure; SI - stroke index,; CI, cardiac index; TPRI, total peripheral index; LVWI, left ventricular work index; LVET, left ventricular ejection time; TFC, thoracic fluid content; LF-RRI, low frequency R-R interval; HF-RRI, high-frequency R-R interval, PSD-RRI, power spectral density R-R interval; LF/HF, ratio between low and high band for heart rate and blood pressure variability; PSD-sBP, power spectral density of systolic blood pressure variability; LF-sBP, low frequency of systolic blood pressure variability; HF-sBP, high frequency of systolic blood pressure variability; PSD-dBP, power spectral density of diastolic blood pressure variability; LF-dBP, low frequency of diastolic blood pressure variability; HF-dBP, high frequency of diastolic blood pressure variability; BRS, baroreflex sensitivity. $^{*}p < 0.05$, $^{**}p < 0.01$.

Table S2. Association of cardiovascular and autonomic measures with RRMS and MG clinical outcomes.

	**RRMS**			**MG**		
	Disease duration	EDSS	Age of first symptom	Disease duration	MGFA	Age of first symptom
delta HR	−0.12	−0.15	−0.30	−0.22	0.15	−0.16
delta sBP	0.12	0.10	0.05	−0.11	0.26	0.04
delta dBP	0.15	0.04	−0.06	−0.04	0.14	−0.12
delta mBP	0.16	0.01	−0.02	−0.07	0.20	−0.03
delta SI	−0.14	0.31	−0.13	−0.10	0.18	0.17
delta CI	−0.18	0.14	−0.16	−0.27	0.13	0.01
delta TPRI	0.22	−0.15	0.28	−0.03	−0.16	0.35
delta LVWI	−0.05	0.17	−0.27	−0.20	0.22	−0.01
delta LVET	−0.04	0.26	0.40	0.02	0.25	−0.02
delta TFC	−0.07	0.01	0.19	−0.16	0.17	0.25

delta LFnu-RRI	−0.25	−0.31	−0.32	−0.10	0.15	−0.27
delta HFnu-RRI	0.25	0.31	0.32	0.10	−0.15	0.27
delta LF-RRI	−0.29	−0.10	−0.12	−0.09	0.21	−0.28
delta HF-RRI	0.07	0.08	0.07	−0.05	0.04	0.36
delta PSD-RRI	−0.09	−0.09	−0.03	−0.13	0.30	0.16
delta LF/HF-RRI	−0.04	−0.39	**−0.42** *	0.03	**0.63** **	0.00
delta LF/HF	−0.02	−0.36	**−0.45** *	0.00	**0.62** **	−0.13
delta LFnu-dBP	−0.08	−0.16	−0.35	−0.10	0.25	−0.38
delta HFnu-dBP	0.31	0.21	0.28	−0.22	−0.09	0.19
delta LF-dBP	−0.08	−0.19	−0.19	0.03	0.11	−0.30
delta HF-dBP	0.14	0.04	0.17	−0.21	−0.15	0.21
delta PSD-dBP	−0.04	0.10	0.17	0.06	0.01	0.15
delta LF/HF-dBP	−0.14	−0.10	−0.34	0.22	0.27	−0.14
delta LFnu-sBP	−0.29	−0.17	−0.25	0.07	0.40	**−0.52** *
delta HFnu-sBP	0.11	0.16	0.40	0.21	0.15	0.16
delta LF-sBP	−0.11	−0.11	−0.12	−0.04	0.15	−0.36
delta HF-sBP	0.17	0.20	**0.43** *	0.05	−0.07	0.15
delta PSD-sBP	0.06	−0.21	−0.31	−0.23	−0.06	−0.07
delta LF/HF-sBP	−0.26	−0.12	**−0.47** *	−0.11	−0.08	**−0.46** *

Delta HR, heart rate; delta sBP, systolic blood pressure; delta dBP, diastolic blood pressure; delta mBP mean blood pressure; delta SI - stroke index; delta CI, cardiac index; delta TPRI, total peripheral index; delta LVWI, left ventricular work index; delta LVET, left ventricular ejection time; delta TFC, thoracic fluid content; delta LF-RRI, low frequency R-R interval; delta HF-RRI, high-frequency R-R interval, delta PSD-RRI, power spectral density R–R interval; delta LF/HF, ratio between low and high band for heart rate and blood pressure variability; delta PSD-sBP, power spectral density of systolic blood pressure variability; delta LF-sBP, low frequency of systolic blood pressure variability; delta HF-sBP, high frequency of systolic blood pressure variability; delta PSD-dBP, power spectral density of diastolic blood pressure variability; LF-dBP, low frequency of diastolic blood pressure variability; delta HF-dBP, high frequency of diastolic blood pressure variability; delta BRS, baroreflex sensitivity; nu, normalised values; delta (change baseline-tilt), statistically significant differences are indicated with *$p < 0.05$, **$p < 0.01$, ***$p < 0.001$.
